# Automated Cerebrospinal Fluid Flow Quantification in Hydrocephalus Using Phase‐Contrast Magnetic Resonance Imaging

**DOI:** 10.1002/nbm.70431

**Published:** 2026-10-26

**Authors:** Eder Alejandro Rodríguez Martínez, Pan Liu, Kimi Owashi, Antoine Gayet, Olivier Balédent

**Affiliations:** ^1^ CHIMERE Laboratory UA21 INSERM University of Picardie Jules Verne Amiens France; ^2^ Institut Faire Faces Amiens France; ^3^ CHU Amiens‐Picardie University Hospital Amiens France; ^4^ Jules Verne Engineering School Amiens France

**Keywords:** cerebrospinal fluid, deep learning, hydrocephalus, interobserver variability, phase‐contrast MRI, segmentation

## Abstract

Quantitative cerebrospinal fluid (CSF) flow analysis with phase‐contrast magnetic resonance imaging depends on segmentation and is affected by operator variability. This cohort method‐comparison study evaluated whether a flow‐aware deep learning (DL) model provides reproducible C2–C3 CSF segmentation and flow quantification compared with repeated manual segmentation. Ten hydrocephalus patients underwent 3‐T phase‐contrast magnetic resonance imaging at C2–C3, with 32 magnitude and phase frames across the cardiac cycle. Three experts and one novice segmented each case twice using semi‐automatic software, generating 80 manual annotations. A pretrained flow‐aware 2D‐UNet generated one fixed automatic segmentation per case. The inference pipeline was deterministic, meaning that repeated processing of the same input with fixed model weights and settings produced identical masks. A multi‐expert reference was defined as the arithmetic mean of the expert measurements. Region of interest (ROI) surface area, peak flow amplitude, and stroke volume were compared using mean difference, Bland–Altman 95% limits of agreement, Lin's concordance correlation coefficient (CCC), and intraclass correlation coefficient (ICC). Expert ICC(1,1) was 0.88–0.97 for surface area, 0.97–1.00 for peak flow amplitude, and 0.97–0.99 for stroke volume; novice values were 0.63, 0.95, and 0.92, respectively. Compared with the multi‐expert reference, model mean differences were −0.42 mm^2^ for surface area, −0.00 mL/s for peak flow amplitude, and −0.01 mL for stroke volume, with CCC values of 0.50, 0.87, and 0.86. Bland–Altman limits of agreement were −69.12 to 68.27 mm^2^, −1.47 to 1.46 mL/s, and −0.34 to 0.31 mL. The model provided rapid, deterministic CSF segmentation with small cohort‐level mean differences in peak flow amplitude and stroke volume; however, individual‐level agreement with the flow‐derived multi‐expert reference was lower than for expert and novice segmentations obtained using the semi‐automatic workflow.

## Graphical abstract

In 10 patients with hydrocephalus, a deterministic deep learning model segmented CSF at C2–C3 on phase‐contrast MRI. Compared with repeated expert and novice segmentations, it produced rapid, consistent results, with small differences in peak flow amplitude and stroke volume relative to a multi‐expert reference.

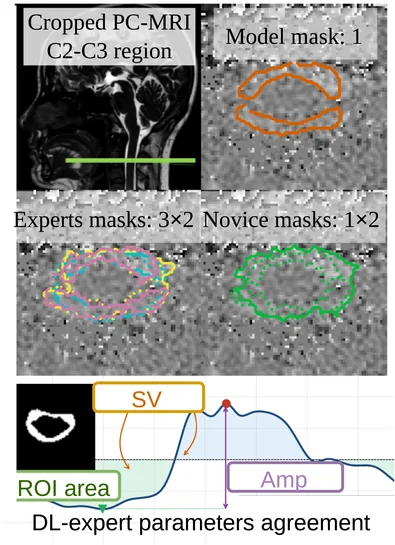

## 1 Introduction

Quantitative assessment of cerebrospinal fluid (CSF) flow is clinically relevant in hydrocephalus because altered craniospinal hydrodynamics may reflect changes in intracranial compliance, ventricular‐subarachnoid pressure transmission, and CSF circulation [1, 2, 3, 4]. Phase‐contrast magnetic resonance imaging (PC‐MRI) provides a noninvasive method for encoding CSF velocity over the cardiac cycle and deriving hydrodynamic end‐points, including flow waveforms, peak flow amplitude, and stroke volume (SV) [5, 6, 7, 8, 9]. These measurements are used to characterize CSF dynamics in neurofluid disorders, but their reliability depends on consistent region‐of‐interest (ROI) delineation and postprocessing.

The cervical C2–C3 level is a relevant region for quantifying CSF dynamics with PC‐MRI because it captures spinal subarachnoid oscillations that are tightly coupled to intracranial blood‐volume changes over the cardiac cycle, and flow‐derived measures at this level—such as peak flow and SV—have shown good reliability and clinical relevance in studies of craniospinal hydrodynamics [10, 11, 12]. However, quantitative analysis at C2–C3 remains demanding because the cervical subarachnoid space has a complex geometry, flow‐sensitive contrast can be limited, and PC‐MRI measurements are vulnerable to partial‐volume effects, eddy‐current‐related background offsets, slice prescription errors, and other sources of variability that influence ROI delineation and downstream flow estimates [11]. In patients, this task may potentially become more challenging when pathology introduces anatomical narrowing or abnormal hydrodynamic patterns, such as altered craniospinal flow, reduced subarachnoid cross‐sectional area, hyperdynamic profiles, or near‐blocked flow, which can increase local irregularity of the signal to be segmented [13]. Recent work suggests that automation of CSF flow analysis in PC‐MRI can be approached through different strategies. In the cerebral aqueduct, Tsou et al. developed a convolutional neural network (CNN) framework for direct automatic segmentation of the aqueduct ROI and showed that CNN‐derived flow parameters could approach expert‐level measurements [14]. At the cervical level, Huang et al. reported a pipeline for spinal CSF flow quantification at C2 in PC‐MRI, using YOLOv4 [15] for region detection followed by independent component analysis for segmentation rather than a full DL segmentation framework [16]. In current practice, CSF quantification at C2–C3 still commonly relies on manual or semi‐automatic segmentation [7, 11, 17]. Existing automation studies have addressed CSF flow analysis in other anatomical regions or with different segmentation strategies, whereas direct DL‐based segmentation at C2–C3 remains insufficiently evaluated.

This study evaluates operator‐dependent variability in C2–C3 CSF segmentation and flow quantification in PC‐MRI, using repeated segmentations from expert and novice operators and comparing them with a fixed automatic DL segmentation pipeline. Specifically, the objective is to assess whether fully repeatable automatic segmentation can provide quantitative CSF flow measurements that are consistent with a multi‐expert reference. The analysis focuses on ROI surface area, flow waveform, peak flow amplitude, and SV, thereby evaluating whether differences in segmentation propagate to clinically relevant hydrodynamic parameters.

## 2 Materials and Methods

This section describes the methodological pipeline used to evaluate automatic and manual CSF segmentation at the C2–C3 level in PC‐MRI. First, PC‐MRI data were acquired in hydrocephalus patients and segmented by human operators and the pretrained flow‐aware DL model. This initial acquisition‐and‐segmentation stage is summarized in Figure 1. Next, the resulting binary masks were converted into quantitative measurements by extracting the ROI surface area, background‐corrected flow waveform, peak flow amplitude, and SV, as illustrated in Figure 2. Finally, these geometric and hydrodynamic parameters were compared across operators and against the multi‐expert reference using complementary repeatability and agreement analyses.

**FIGURE 1:**
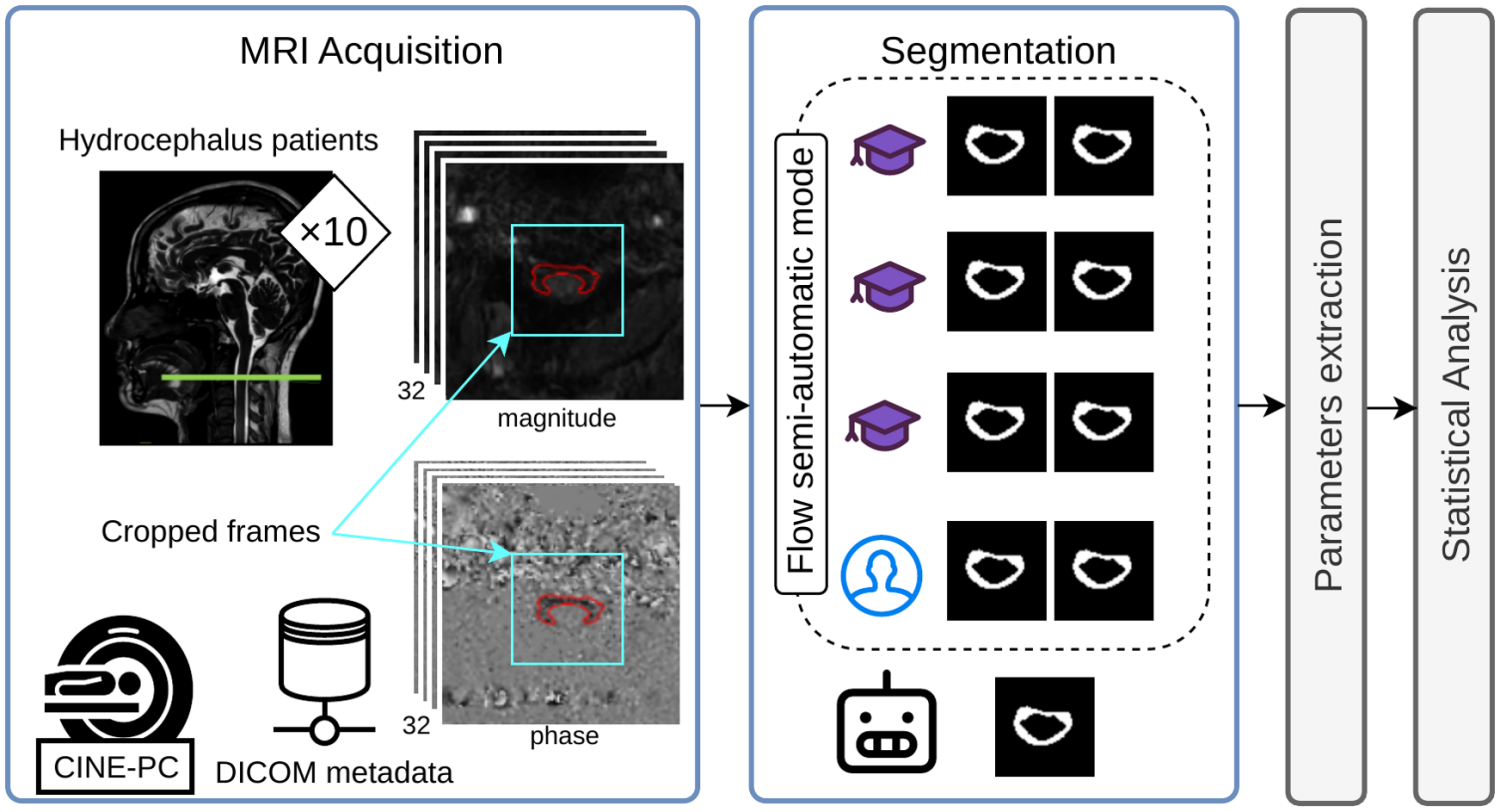
MRI acquisition and segmentation. Phase‐contrast MRI was acquired at the C2–C3 level. Human segmentation masks were obtained using the semi‐automatic flow software, and the DL model processed centrally cropped 80 × 80‐pixel images.

**FIGURE 2:**
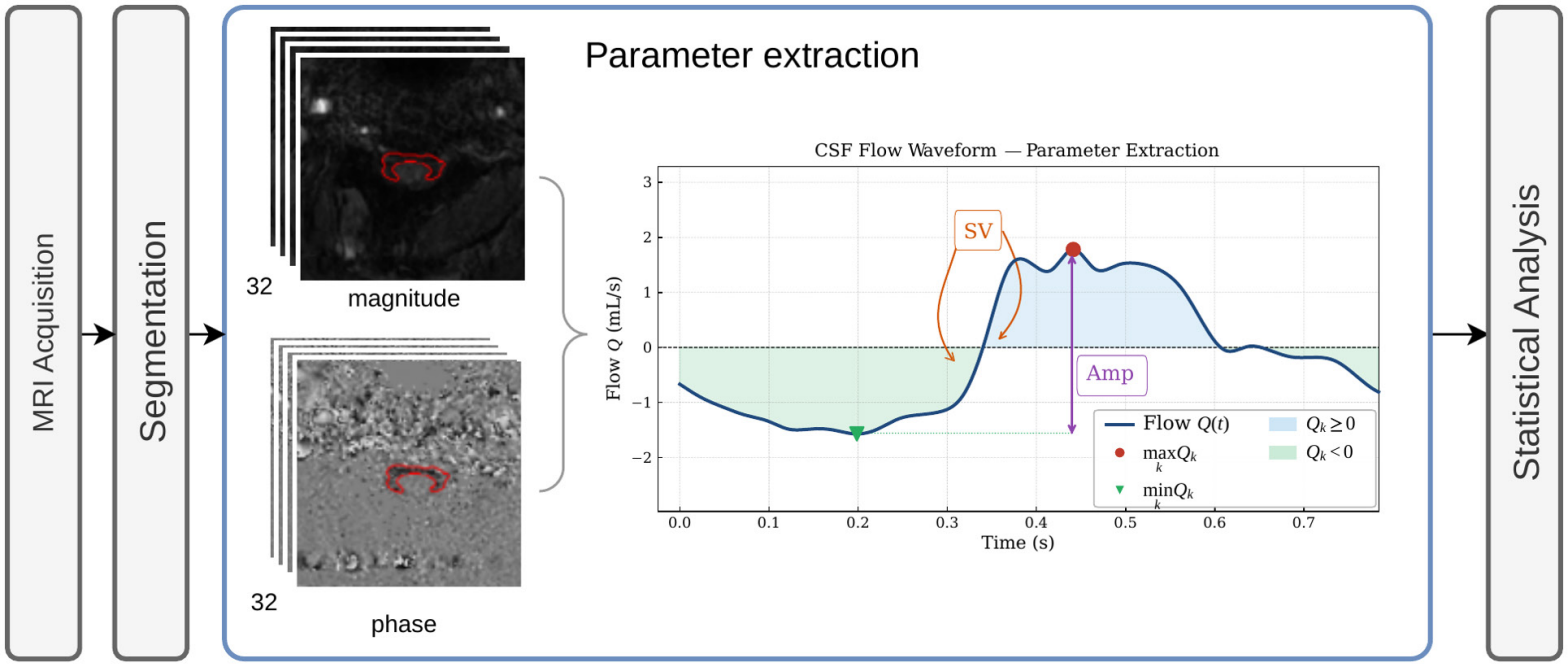
Parameter extraction. Red contours delineate the cerebrospinal fluid (CSF) region of interest at C2–C3 on the magnitude and phase images (32 cardiac frames each). Segmentation masks and background‐corrected, phase‐derived velocities were used to obtain the CSF flow waveform. Peak flow amplitude (Amp) is the difference between maximum and minimum flow, indicated by the purple arrow. Stroke volume (SV) is the integral of absolute flow over the cardiac cycle, represented by the total shaded area above and below zero.

### 2.1 MRI Acquisition

The study was conducted in accordance with the Declaration of Helsinki and approved by the Ethics Review Committee (ID‐RCB: 2021‐A00240‐41). All participants provided written informed consent prior to inclusion.

The cohort used for inter‐ and intra‐rater variability consisted of 10 patients diagnosed with hydrocephalus whose ages ranged from 37 to 87 years old with a mean of 68.8 and a sex distribution of six men and four women. No patients were excluded.

All patients underwent PC‐MRI targeting the C2–C3 cervical level on a 3‐T Philips Achieva scanner (Amiens, France). The sequence was parameterized as follows: velocity encoding (𝑣enc) is set to 5 or 10 cm/s depending on aliasing, in‐plane pixel resolution of 0.8 × 0.8 mm^2^, echo time (TE) was 7.8 or 8.8 ms, repetition time (TR) was 12.7 or 13.6 ms, sensitive encoding (SENSE) factor of 1.5, slice thickness of 2 mm, and a flip angle of 30°. The sampling time ranged from 68.64 to 119.44 s (mean: 87.217 s). Thirty‐two frames (magnitude and phase images) were reconstructed across the cardiac cycle to capture the full pulsatile dynamics of the CSF. Specifically, cardiac synchronization was performed using peripheral pulse plethysmography with retrospective cardiac gating. Participants breathed freely and spontaneously throughout the acquisition. No respiratory signal was recorded, and no respiratory gating or respiratory‐motion compensation was applied. Therefore, the 32 reconstructed phases represent an average cardiac cycle retrospectively reconstructed from multiple cardiac cycles acquired at uncontrolled and varying respiratory phases. Respiratory‐driven CSF motion was not separately resolved or quantified in the present analysis. Lastly, a fixed 80 × 80‐pixel window centered on the original 240 × 240 images was applied to all magnitude and phase frames and the corresponding segmentation masks exported by the flow software. The crop size was selected empirically to retain the complete C2–C3 CSF ROI while reducing the input dimensions and computational requirements. Cropping preserved the original pixel spacing without resampling. During model development, all cropped masks were visually inspected to confirm complete ROI inclusion before the dataset was split. Figure 1 displays this process in the left block.

### 2.2 Segmentation

The C2–C3 cervical region was segmented in the 10 hydrocephalus patients by four human operators and by one pretrained flow‐aware 2D U‐Net model, originally proposed in [18]. The DL model received the centrally cropped 80 × 80‐pixel PC‐MRI frames as input. Three experts—one medical imaging faculty member with 30 years of experience and two neuroscience researchers with 9 and 5 years of experience in PC‐MRI analysis—and one novice operator with 3 h of experience segmented each case twice using the semi‐automatic mode of the flow software. Although none of the experts were radiologists, they had extensive experience in cervical PC‐MRI analysis, CSF‐flow quantification, and the relevant spinal anatomy. The two segmentation sessions were separated by at least 1 day, generating two masks per case. Briefly, the adapted 2D U‐Net comprised four downsampling stages and a bottleneck, processing an 80 × 80 input formed by concatenating 32 phase and 32 magnitude frames (64 channels). The encoder used 32, 64, 128, 256, and 512 feature channels, reaching a minimum resolution of 5 × 5 pixels. Each stage contained two 3 × 3 convolutions with batch normalization and ReLU activation; the decoder used transposed convolutions and skip connections, followed by a 1 × 1 output convolution. Its application‐specific adaptations were the full‐cycle multichannel input and flow‐aware loss. The fixed weights were trained on 43 healthy volunteers and 43 hydrocephalus patients using Adam for up to 60 epochs with combined Dice, binary cross‐entropy, and flow‐consistency losses. No fine‐tuning or model selection was performed on the present cohort. The DL model was used as a fixed automatic comparator in this operator‐variability study rather than introduced as the primary methodological contribution. Because the inference pipeline was deterministic, meaning that repeated processing of the same input with fixed model weights and settings produced identical masks, the DL model generated one segmentation per case. The source code and model weights are provided in the public repository to support reproducibility.

### 2.3 Parameters Extraction

From the segmented CSF region, four parameters were extracted from the PC‐MRI data: ROI surface area (in mm^2^), flow waveform (in mL/s), peak flow amplitude (in mL/s), and SV (in mL). The conversion from binary segmentation to quantitative hydrodynamic measurements is illustrated in Figure 2 and summarized next. For each segmentation mask, the CSF ROI was used to compute the ROI surface area and the mean phase‐derived velocity after background correction with a nearby static‐tissue reference region [19]. These quantities were combined to obtain the CSF flow waveform over the cardiac cycle, from which peak flow amplitude and SV were derived.

Let *Ω* denote the padded binary mask (at original resolution of 240 × 240) of the CSF region and let ∆𝑥∆𝑦 be the in‐plane pixel area in mm^2^. The ROI surface area was computed as follows:

(1) $$A=\left|\Omega \right|\mathrm{\Delta x\Delta}$$

where |*Ω*| is the number of pixels inside the mask.

The mean velocity within the ROI was obtained from the phase image after background correction using a static‐tissue reference region:

(2) $${\underline{v}}_{k}={\underline{v}}_{k}^{\text{measured}}-{\underline{v}}_{k}^{\mathrm{ref}},$$

where 𝑘 is the frame index, ${\underline{v}}_{k}^{\text{measured}}$ is the mean velocity measured within the segmented CSF region at frame 𝑘, and ${\underline{v}}_{k}^{\mathrm{ref}}$ is the mean velocity measured in a nearby static‐tissue reference region, used to estimate the background phase offset and correct residual phase bias.

The flow waveform (Figure 2) was then computed as follows:

(3) $$Q_{k}=A{\underline{v}}_{k}.$$

Peak flow amplitude was defined as follows:

(4) $$\mathrm{Amp}=Q_{\max}-Q_{\min}.$$

SV was defined as the total oscillatory volume over a cardiac cycle and estimated from the flow waveform:

(5) $$\mathrm{SV}=\Sigma \left|Q\right|\mathrm{\Delta t}$$

where ∆𝑡 is the temporal interval between two consecutive cardiac samples from a total of 32. For numerical integration, the flow waveform was first resampled by cubic spline interpolation to a temporal resolution 10 times higher than the original one, and stroke volume was then computed from the interpolated waveform. All parameters were computed for each manual annotation and for the DL prediction.

### 2.4 Statistical Analysis

Statistical analysis was designed to evaluate both measurement repeatability and agreement for the three study endpoints: ROI surface area, peak flow amplitude, and SV. Group‐level results were summarized in physical units as mean ± standard deviation (SD). In addition, mean difference relative to the reference was reported to quantify systematic overestimation or underestimation. For each case, a multi‐expert reference was defined as the average of the six expert measurements, obtained from three experts who each segmented the case twice. Agreement of each operator and of the DL model with these reference values was assessed separately for each parameter. The novice operator was included in the same framework as an additional human benchmark.

Repeatability was evaluated for each human operator from the two repeated segmentation sessions using the intraclass correlation coefficient, ICC(1,1) [11, 20]. For the DL model, repeated session variability was not expected because the method is deterministic.

Agreement with the multi‐expert reference was assessed using complementary statistics: Mean difference was computed to estimate systematic bias relative to the reference; Bland–Altman analysis was used to quantify disagreement in the original units of each parameter, and 95% limits of agreement (LoA) were reported as mean difference ± 1.96 × SD of the paired differences; Lin's concordance correlation coefficient (CCC) was computed to evaluate numerical concordance [21, 22]. For the nine comparisons shown in Figure 4, mean method‐minus‐reference differences were tested against zero using two‐sided one‐sample *t*‐tests on the 10 patient‐level differences (9 degrees of freedom). Unadjusted 95% confidence intervals for the mean differences were calculated using the *t*‐distribution, and *p*‐values were adjusted using the Holm procedure across the nine mean‐bias tests.

To explore whether disagreement varied with measurement magnitude, ordinary least‐squares regression with an intercept was fitted to each Bland–Altman panel, using the method‐minus‐reference difference as the outcome and their pairwise mean as the predictor. Human endpoint values were averaged across the two sessions within each patient, yielding 10 observations per panel. Slopes were reported with unadjusted 95% confidence intervals, and two‐sided *p*‐values were adjusted using the Holm procedure across the nine combinations of three endpoints and three methods.

Repeated expert delineations were used to estimate repeatability using ICC, and each operator and the DL model were compared against the multi‐expert reference using mean difference, LoA, and CCC. Furthermore, three cases were selected according to low, medium, and high expert ROI surface variation.

## 3 Results

All 10 samples were validated and included in the analysis. Manual segmentation with the semi‐automatic workflow required approximately 1–3 min per delineation, whereas the DL model produced one segmentation per subject in less than 1 s. Table 1 summarizes the quantitative results reported in this section.

**TABLE 1:** Parameter statistics for individual measurements from each operator and the DL model.

Parameter	Operator	Mean ± SD	Mean difference	95% LoA	CCC	ICC(1,1)
ROI surface (mm^2^)	Reference	110 ± 33	0.00	—	—	—
	Expert 1	105 ± 29	−5.70	−24.0 to 12.62	0.94	0.88
	Expert 2	114 ± 39	3.54	−15.66 to 22.74	0.96	0.97
	Expert 3	112 ± 31	2.16	−14.08 to 18.40	0.96	0.93
	Novice	117 ± 34	6.95	−31.31 to 45.20	0.81	0.63
	Model	110 ± 37	−0.42	−69.12 to 68.27	0.5	N/A
Peak flow amplitude (mL/s)	Reference	3.3 ± 1.3	0.00	—	—	—
	Expert 1	3.2 ± 1.3	−0.12	−0.49 to 0.25	0.99	0.97
	Expert 2	3.4 ± 1.3	0.10	−0.15 to 0.36	0.99	0.99
	Expert 3	3.3 ± 1.3	0.01	−0.28 to 0.30	0.99	1.00
	Novice	3.4 ± 1.4	0.13	−0.54 to 0.80	0.96	0.95
	Model	3.3 ± 1.6	0.00	−1.47 to 1.46	0.87	N/A
SV (mL)	Reference	0.7 ± 0.3	0.00	—	—	—
	Expert 1	0.7 ± 0.3	−0.03	−0.11 to 0.06	0.98	0.97
	Expert 2	0.8 ± 0.3	0.03	−0.03 to 0.08	0.99	0.99
	Expert 3	0.7 ± 0.3	0.00	−0.06 to 0.06	0.99	0.99
	Novice	0.8 ± 0.3	0.02	−0.13 to 0.18	0.95	0.92
	Model	0.7 ± 0.3	−0.01	−0.34 to 0.31	0.86	N/A

*Note:* Mean difference and LoA belong to the Bland–Altman test.

Abbreviations: CCC, concordance correlation coefficient; ICC, intraclass correlation coefficient; LoA, limits of agreement; ROI, region of interest; SD, standard deviation; SV, stroke volume.

### 3.1 Repeatability Across Operators

Across the three experts, ICC(1,1) values ranged from 0.88 to 0.97 for ROI surface area, from 0.97 to 1.00 for peak flow amplitude, and from 0.97 to 0.99 for SV. The novice showed lower repeatability than the experts for all three parameters, with ICC values of 0.63 for ROI surface area, 0.95 for peak flow amplitude, and 0.92 for SV. Because the DL inference pipeline was deterministic, repeated processing of the same input produced identical masks; therefore, ICC was not estimated for the DL model and is reported as not applicable (N/A).

### 3.2 Agreement With the Multi‐Expert Reference Values

The multi‐expert reference values ranged from 67.0 to 173.3 mm^2^ for ROI surface area, from 1.198 to 4.928 mL/s for peak flow amplitude, and from 0.354 to 1.220 mL for SV. Mean reference values were 110 ± 33 mm^2^ for ROI surface area, 3.3 ± 1.3 mL/s for peak flow amplitude, and 0.7 ± 0.3 mL for SV. Figure 3 illustrates the spatial manifestation of segmentation variability across repeated expert delineations for a representative case.

**FIGURE 3:**
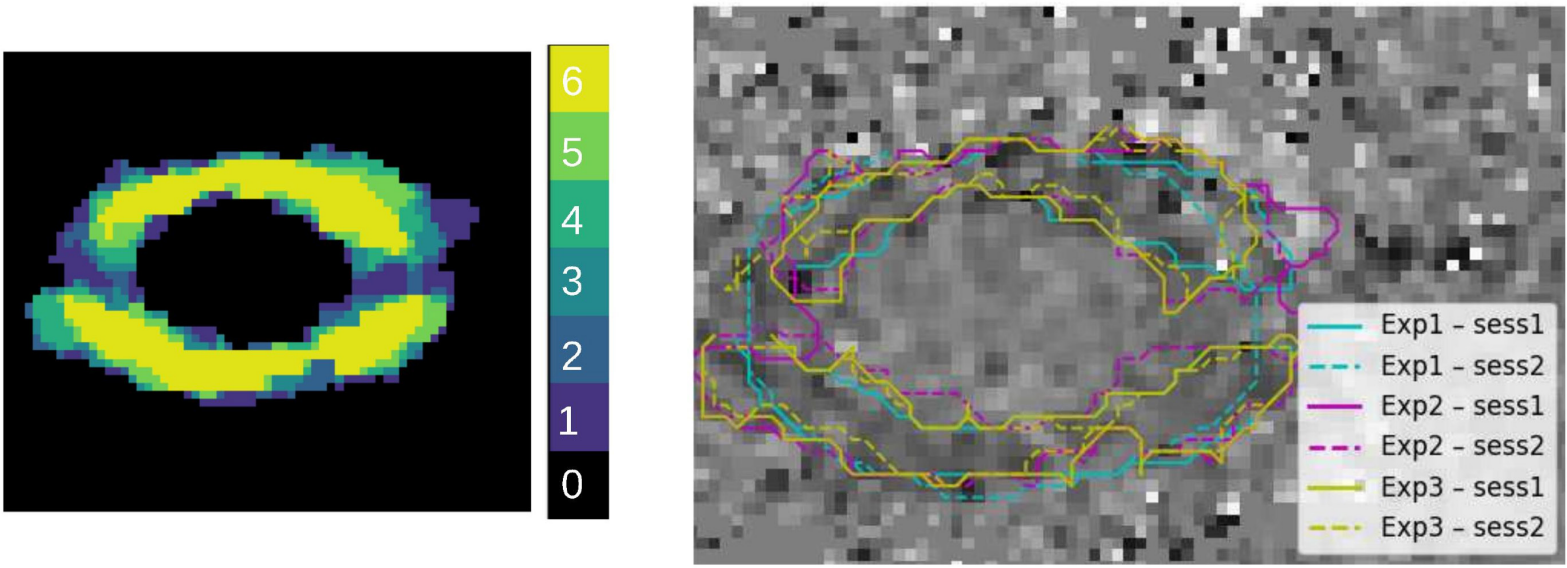
Spatial variability of expert segmentations for a representative case. Left: expert consensus map obtained from the six expert segmentations. Right: overlay of the six expert contours on the phase image.

For ROI surface area, expert mean values in Table 1 ranged from 105 to 114 mm^2^, whereas the novice and DL model yielded 117 ± 34 and 110 ± 37 mm^2^, respectively. At the case level, the novice ROI surface ranged from 76.2 to 176.0 mm^2^, whereas the model ROI surface ranged from 39.8 to 153.8 mm^2^. Relative to the reference, expert mean differences ranged from −5.70 to 3.54 mm^2^, with CCC values from 0.94 to 0.96. The novice showed a mean difference of 6.95 mm^2^, limits of agreement from −31.31 to 45.20 mm^2^, and CCC of 0.81. The DL model showed a mean difference of −0.42 mm^2^, limits of agreement from −69.12 to 68.27 mm^2^, and CCC of 0.50.

For peak flow amplitude, expert mean values ranged from 3.2 to 3.4 mL/s, whereas the novice and DL model yielded 3.4 ± 1.4 and 3.3 ± 1.6 mL/s, respectively. At the case level, novice peak flow amplitude ranged from 1.18 to 5.34 mL/s, whereas model peak flow amplitude ranged from 0.93 to 5.55 mL/s. Relative to the multi‐expert reference values, expert mean differences ranged from −0.12 to 0.10 mL/s, and all expert CCC values were 0.99. The novice showed a mean difference of 0.13 mL/s, limits of agreement from −0.54 to 0.80 mL/s, and CCC of 0.96. The DL model showed a mean difference of −0.00 mL/s, limits of agreement from −1.47 to 1.46 mL/s, and CCC of 0.87.

For SV, expert mean values ranged from 0.7 to 0.8 mL, whereas the novice and DL model yielded 0.8 ± 0.3 and 0.7 ± 0.3 mL, respectively. At the case level, novice SV ranged from 0.38 to 1.26 mL, whereas model SV ranged from 0.28 to 1.34 mL. Relative to the reference, expert mean differences ranged from −0.03 to 0.03 mL, with CCC values from 0.98 to 0.99. The novice showed a mean difference of 0.02 mL, limits of agreement from −0.13 to 0.18 mL, and CCC of 0.95. The DL model showed a mean difference of −0.01 mL, limits of agreement from −0.34 to 0.31 mL, and CCC of 0.86.

Figure 4 shows the Bland–Altman agreement analysis for the DL model, Expert 1 and novice relative to the multi‐expert reference, with both sessions averaged for Expert 1 and the novice. The solid horizontal line indicates the mean difference, and the dashed lines indicate the 95% limits of agreement. No mean difference was statistically significant after Holm correction (all adjusted *p* ≥ 0.0704; Table S2). Expert 1 showed negative mean differences for all three biomarkers that were significant before correction.

**FIGURE 4:**
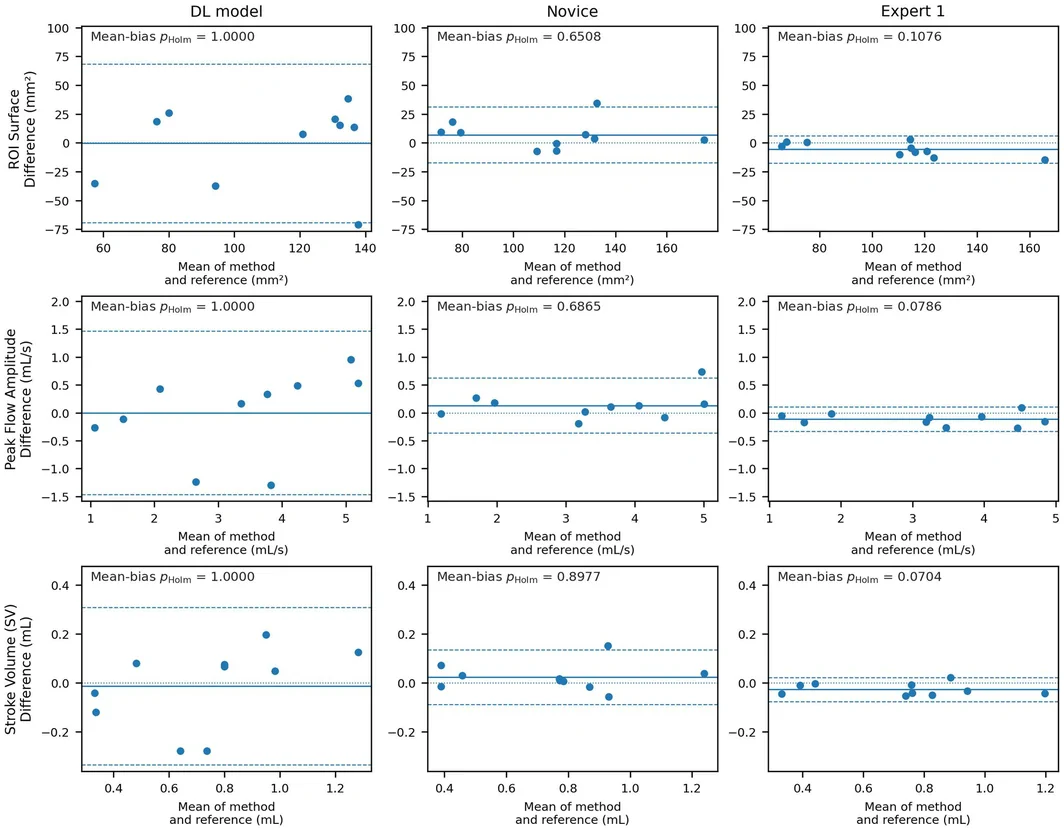
Bland–Altman analysis of agreement with the multi‐expert reference. Agreement of the novice operator, Expert 1 and DL model with the multi‐expert reference values for region‐of‐interest surface area, peak flow amplitude, and stroke volume. For the novice operator and Expert 1, both sessions were averaged. Annotated *p*‐values assess whether the mean method‐minus‐reference difference differs from zero using two‐sided one‐sample *t*‐tests on the 10 patient‐level differences, with Holm correction across the nine panels. No mean bias was statistically significant after correction at α = 0.05. The solid horizontal lines indicate mean differences, and the dashed lines indicate the 95% limits of agreement.

The estimated DL slopes were positive for ROI surface area, peak flow amplitude, and SV (0.150, 0.204, and 0.249, respectively), but all corresponding 95% confidence intervals included zero (Table S1). No linear trend reached statistical significance after Holm correction across the nine panels. Expert 1 showed a negative slope for ROI surface area that was significant before correction (*p* = 0.0184) but not after correction (adjusted *p* = 0.1652).

### 3.3 Representative Cases Across Disagreement Levels

Three representative cases were selected to illustrate low, medium, and high variation in their expert ROI surface variation. In Figure 5, the selected cases are shown in parameter space together with expert measurements, their mean and dispersion, and the corresponding DL and novice measurements. The pixel maps encode expert support and agreement with the DL segmentation. Black indicates pixels selected by neither the experts nor the DL model; orange indicates pixels selected only by the DL model. Blue indicates pixels selected by both the DL model and at least one expert, whereas red indicates pixels selected by at least one expert but omitted by the DL model. Within the blue and red scales, color intensity increases with the number of expert selections, from one to six. Figure 6 illustrates the same cases by showing the DL model, expert‐mask contour (pixels selected at least three times by the experts) and one novice contour over three phase frames. For each case, the expert consensus map is also shown, together with the flow waveforms computed from the DL segmentation, one novice segmentation, the expert‐vote mask, and the expert‐intersection mask.

**FIGURE 5:**
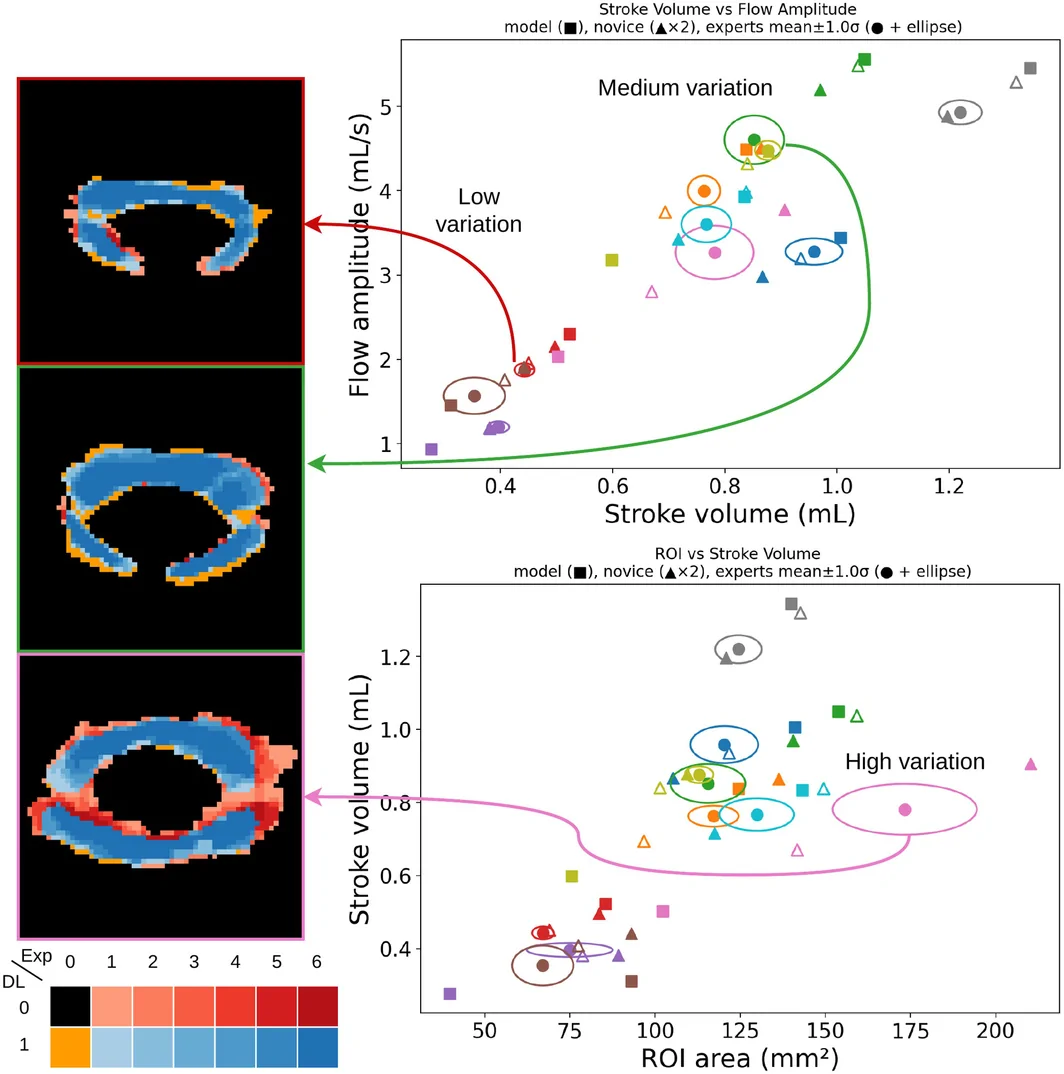
Representative cases in parameter space. Left: discrete expert‐support and DL‐agreement map (blue: agreement with experts and DL, red: expert‐selected pixel omitted by the DL model, orange: pixel selected by the DL model only). Right: cases represented in parameter space, showing expert measurements, their mean and dispersion ellipse (at one standard deviation).

**FIGURE 6:**
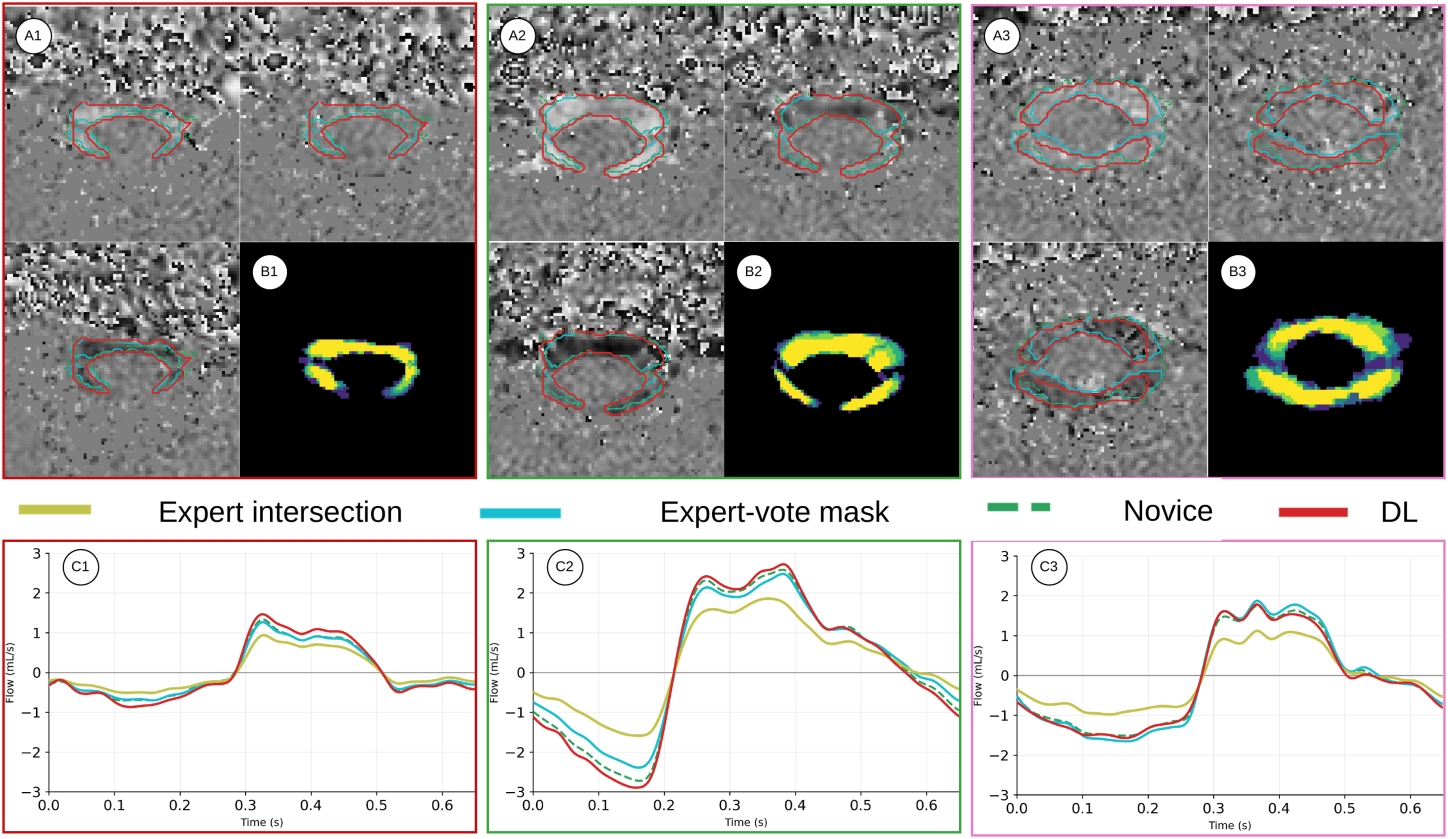
Representative cases and their flow waveforms. (A) DL segmentation, expert‐vote mask (defined by at least three expert votes), and one novice segmentation (Session 1) contours are shown over three phase frames. (B) Expert consensus map. (C) flow waveforms computed from the DL segmentation, one novice segmentation, the expert‐vote mask, and the expert‐intersection mask (defined by the pixels selected in all six expert annotations).

For the easy case, the reference values were 67.1 ± 3.2 mm^2^ for ROI surface area, 1.875 ± 0.081 mL/s for peak flow amplitude, and 0.443 ± 0.067 mL for SV. The DL model yielded 93.3 mm^2^, 2.330 mL/s, and 0.548 mL, whereas the novice yielded 76.2 ± 10.3 mm^2^, 2.056 ± 0.144 mL/s, and 0.480 ± 0.078 mL.

For the medium case, the reference values were 115.5 ± 11.0 mm^2^ for ROI surface area, 4.60 ± 0.29 mL/s for peak flow amplitude, and 0.853 ± 0.053 mL for SV. The DL model yielded 153.8 mm^2^, 5.555 mL/s, and 1.037 mL, whereas the novice yielded 149.9 ± 13.3 mm^2^, 5.311 ± 0.204 mL/s, and 0.975 ± 0.051 mL.

For the hard case, the reference values were 173.3 ± 21.2 mm^2^ for ROI surface area, 3.267 ± 0.317 mL/s for peak flow amplitude, and 0.782 ± 0.069 mL for SV. The DL model yielded 102.2 mm^2^, 2.031 mL/s, and 0.503 mL, whereas the novice yielded 176.0 ± 48.4 mm^2^, 3.289 ± 0.687 mL/s, and 0.789 ± 0.167 mL.

## 4 Discussion

This study evaluated whether automatic segmentation at the C2–C3 level can support reliable quantification of CSF dynamics in PC‐MRI beyond contour extraction alone. The DL model produced deterministic segmentations in less than 1 s, compared with approximately 1–3 min per delineation using the human‐operated semi‐automatic workflow. For peak flow amplitude and SV, cohort‐level mean differences relative to the multi‐expert reference were small. Nevertheless, the model showed wider limits of agreement and lower CCC values than both the experts and the novice across all three endpoints, indicating that individual‐level interchangeability with the evaluated semi‐automatic workflow was not established. Small mean differences can result from cancellation of positive and negative discrepancies and should therefore be interpreted as limited cohort‐level systematic offset rather than as evidence of close agreement for individual patients. Reducing variability in these parameters is relevant not only at the operator level but also across laboratories, scanners, and postprocessing pipelines, because inconsistent quantification can complicate comparison between studies, limit reproducibility, and weaken the reliability of flow‐derived parameters [7, 23].

Although the DL slope estimates were positive, their wide confidence intervals and the small sample size limit conclusions about magnitude‐dependent disagreement. Therefore, the nonsignificant results should not be interpreted as demonstrating the absence of proportional bias.

A plausible explanation for the observed dissociation between geometric and hydrodynamic agreement is that not all pixels contribute equally to flow quantification. In PC‐MRI, boundary pixels are more vulnerable to partial‐volume effects and other local inaccuracies, whereas the central portion of the segmented CSF region tends to contribute more stably to the mean velocity and resulting waveform [24, 25]. Under this interpretation, moderate disagreement at the contour level may have a limited impact on peak flow amplitude and SV when the central flow‐carrying region is captured consistently. This mechanism may contribute to the higher CCC values observed for peak flow amplitude and SV (0.87 and 0.86, respectively) than for ROI surface area (0.50), although its contribution was not directly evaluated.

These comparisons should also be interpreted in light of the reference construction and the shared human segmentation workflow. No independent anatomical ground truth was available. The multi‐expert reference was calculated from measurements derived from expert segmentations generated using the same semi‐automatic flow workflow used for the human measurements, and each expert's annotations contributed to the reference against which that expert was evaluated, potentially favoring expert–reference agreement. Neither the novice nor the DL model contributed to the reference. In addition, Figure 4 compares human measurements averaged across two sessions with a single deterministic DL output; averaging may narrow the human limits of agreement relative to a single delineation.

The shared flow software and semi‐automatic segmentation workflow may also promote common segmentation conventions among the human operators, including the novice. Previous studies demonstrated that semi‐automatic segmentation can reduce interobserver variability and affect the resulting ROI and flow measurements [26, 27]. Therefore, the wider limits of agreement observed for the DL model should be interpreted as lower agreement with this flow‐derived multi‐expert reference rather than as direct evidence of lower absolute anatomical accuracy. Conversely, the present study cannot establish that the DL segmentation is more accurate than the flow‐based measurements because no independent ground truth was available. The DL model may itself reflect segmentation conventions inherited from its training annotations, which were also generated using flow. An independent reference standard would therefore be required to determine to what extent the observed disagreement reflects DL segmentation error, shared flow‐based segmentation conventions, or both.

The interest of this problem is reinforced by the physiological relevance and technical difficulty of the C2–C3 region. Cervical CSF oscillations are closely linked to craniospinal hydrodynamics and to the interaction between CSF and blood‐volume changes over the cardiac cycle [8, 28]. At the same time, segmentation at this level remains challenging because of the complex geometry of the cervical subarachnoid space, limited flow‐sensitive contrast, background phase offsets, and sensitivity to partial‐volume and acquisition‐related effects. In pathological cases, these difficulties may be further amplified by anatomical narrowing or altered flow patterns.

Our results also help position this work relative to previous automation efforts. Prior studies have reported automatic analysis in the cerebral aqueduct and AI‐assisted pipelines at the cervical level [14, 16], but the present work focuses specifically on direct DL‐based segmentation at C2–C3 and evaluates it against repeated expert and novice annotations using both geometric and hydrodynamic endpoints. In this sense, the contribution is not limited to replacing manual contouring, but to testing whether a flow‐aware DL model can preserve the quantities that matter most for downstream CSF analysis while eliminating intra‐operator variability.

The representative cases further clarify the behavior of the DL model. In low variation cases, the DL contours may differ from the expert segmentations while still preserving the overall waveform and derived parameters reasonably well. In high variation cases, however, the geometric deviation becomes large enough to induce relevant underestimation of ROI surface area, peak flow amplitude, and SV. These examples indicate that the method is not uniformly robust across all morphologies and flow conditions. They also suggest that local anatomical complexity, reduced contrast, or abnormal hydrodynamic configurations remain important failure modes for automatic segmentation.

## 5 Limitations

This study has some limitations. First, the cohort was small and restricted to hydrocephalus cases from a limited acquisition setting; broader validation on larger, multicenter datasets spanning wider anatomical and pathological variability is therefore necessary. Second, the DL model showed limited robustness in some difficult examples; this motivates further refinement of training data diversity, robustness evaluation across scanners and protocols, and assessment of generalization under more heterogeneous conditions. Third, the present analysis was restricted to cardiac‐phase‐resolved CSF motion. Because respiration was neither monitored nor controlled, respiratory‐driven CSF motion could not be separately assessed [29]. The retrospectively gated 32‐phase waveform represents cardiac‐synchronized CSF dynamics averaged across multiple cardiac cycles acquired at varying and uncontrolled respiratory phases [29]. Respiratory‐related fluctuations were therefore expected to be partly averaged during cardiac‐cycle reconstruction. However, respiration can modulate spinal CSF velocity, flow amplitude, and displacement, and residual respiratory effects on the reconstructed waveform cannot be excluded [29, 30, 31]. Consequently, the present results should be interpreted as reflecting average cardiac‐synchronized CSF dynamics rather than the full cardiorespiratory variability of spinal CSF motion. Peripheral pulse gating provides straightforward sensor placement and optical detection that avoids the magnetohydrodynamic distortion that can affect ECG recordings in MRI [32]. However, peripheral pulse detection is delayed relative to central cardiac events, and no additional correction for this delay was applied during postprocessing in our study. Consequently, interpretation of absolute cardiac–CSF timing relationships is limited, and CSF timing relative to the ECG or central arterial pulse was not assessed. Furthermore, beat‐to‐beat variability in trigger timing can affect waveform reconstruction [33]. In this respect, retrospective ECG gating has shown greater quantitative and temporal precision for spinal CSF flow measurements at 3 T [34]. Although all segmentation methods in our study used the same reconstructed acquisitions, this shared acquisition basis does not exclude gating‐related effects on flow amplitude and SV, which were not independently assessed. Finally, because the DL model was deployed as a fixed, pretrained comparator without fine‐tuning on the present cohort, there remains significant potential to improve segmentation performance. Optimizing the U‐Net architecture or its training parameters for domain‐specific anatomical variations could yield better geometric recovery, particularly for the high‐variation cases where local anatomical complexity currently limits robustness.

## 6 Conclusion

This study showed that a flow‐aware DL model can provide fast, deterministic CSF segmentation with small cohort‐level differences in flow‐derived measurements. Agreement with the flow‐derived multi‐expert reference was lower at the individual level, although this reference was not an independent ground truth, and human segmentations were obtained using the same semi‐automatic flow workflow. The results support automatic segmentation as a practical approach to reduce operator‐dependent variability, whereas broader validation remains necessary.

## Author Contributions

**Eder Alejandro Rodríguez Martínez:** conceptualization, formal analysis, methodology, visualization, writing. **Pan Liu:** investigation, software, supervision, validation. **Kimi Owashi:** investigation, validation. **Antoine Gayet:** data curation, visualization. **Olivier Balédent:** funding acquisition, investigation, project administration, resources, supervision.

## Funding

This work was supported by the European Regional Development Fund under the Grant Number 21004127; Conseil Régional de Picardie; Fondation des Gueules Cassées (55‐2022); FiGuRES EquipEX (ANR‐10‐EOPX‐01‐01); and Immouv/ROBOT.

## Conflicts of Interest

The authors declare no conflicts of interest.

## Supporting information


**Table S1:** Linear trends in Bland–Altman comparisons with the multi‐expert reference.

**Table S2:** Mean differences and tests of bias in Bland–Altman comparisons with the multi‐expert reference.

## Data Availability

The data that support the findings of this study are openly available in Experts‐vs‐2D‐UNet‐C2‐C3‐segment at https://github.com/eder1234/Experts‐vs‐2D‐UNet‐C2‐C3‐segmentation.
