# Supplementary material for: Automated Cerebrospinal Fluid Flow Quantification in Hydrocephalus Using Phase‐Contrast Magnetic Resonance Imaging
Source: NMR Biomed. 2026 Oct 26;39(12):e70431. doi: 10.1002/nbm.70431 (PMC13697231; doi:10.1002/nbm.70431)
Supplement: Supplementary file 1 [file NBM-39-e70431-s001.docx]

**Supporting Information**

Automated Cerebrospinal Fluid Flow Quantification in Hydrocephalus Using Phase-Contrast Magnetic Resonance Imaging

**Table S1.** Linear trends in Bland–Altman comparisons with the multi-expert reference.

| **Biomarker** | **Method** | **Slope** | **SE** | **Unadjusted 95% CI** | **Pearson *r*** | **Unadjusted *p*** | **Holm-adjusted *p*** |
| --- | --- | --- | --- | --- | --- | --- | --- |
| ROI surface area | DL model | 0.1495 | 0.4077 | [−0.7907, 1.0897] | 0.1286 | 0.7234 | 1.0000 |
|  | Novice | −0.0424 | 0.1371 | [−0.3586, 0.2737] | −0.1088 | 0.7648 | 1.0000 |
|  | Expert 1 | −0.1425 | 0.0483 | [−0.2537, −0.0312] | −0.7221 | 0.0184 | 0.1652 |
| Peak flow amplitude | DL model | 0.2037 | 0.1713 | [−0.1914, 0.5988] | 0.3875 | 0.2686 | 1.0000 |
|  | Novice | 0.0530 | 0.0630 | [−0.0923, 0.1983] | 0.2850 | 0.4248 | 1.0000 |
|  | Expert 1 | −0.0107 | 0.0299 | [−0.0797, 0.0583] | −0.1250 | 0.7308 | 1.0000 |
| Stroke volume | DL model | 0.2488 | 0.1726 | [−0.1492, 0.6468] | 0.4541 | 0.1874 | 1.0000 |
|  | Novice | 0.0063 | 0.0739 | [−0.1641, 0.1768] | 0.0302 | 0.9339 | 1.0000 |
|  | Expert 1 | −0.0141 | 0.0324 | [−0.0889, 0.0606] | −0.1523 | 0.6744 | 1.0000 |

**Analysis.** Ordinary least-squares regression with an intercept was fitted separately to each of the nine panels in Figure 4. The outcome was the method-minus-reference difference, and the predictor was the mean of the method value and the multi-expert reference value. Each regression included 10 patients, with one observation per patient. Slopes and their standard errors are dimensionless. Pearson’s r describes the association between the differences and paired means.

**Measurements.** For the novice and Expert 1, endpoint values were averaged across the two segmentation sessions within each patient; the DL model was evaluated once. For each patient and endpoint, the multi-expert reference was used (three experts, two sessions each). Expert 1 contributed to this reference.

**Statistical reporting.** Confidence intervals are unadjusted 95% intervals. Unadjusted p-values are from two-sided tests of a zero slope with 8 degrees of freedom. Holm adjustment covers all nine tests. No test reached statistical significance after adjustment at α = 0.05; this does not establish the absence of magnitude-dependent disagreement.

**Abbreviations.** CI, confidence interval; DL, deep learning; ROI, region of interest; SE, standard error.
