# Supplementary material for: Automated Cerebrospinal Fluid Flow Quantification in Hydrocephalus Using Phase‐Contrast Magnetic Resonance Imaging
Source: NMR Biomed. 2026 Oct 26;39(12):e70431. doi: 10.1002/nbm.70431 (PMC13697231; doi:10.1002/nbm.70431)
Supplement: Supplementary file 2 [file NBM-39-e70431-s002.docx]

**Supporting Information**

Automated Cerebrospinal Fluid Flow Quantification in Hydrocephalus Using Phase-Contrast Magnetic Resonance Imaging

**Table S2.** Mean differences and tests of bias in Bland–Altman comparisons with the multi-expert reference.

| **Biomarker** | **Method** | **Mean bias** | **Unadjusted 95% CI for mean bias** | **Unadjusted *p*** | **Holm-adjusted *p*** |
| --- | --- | --- | --- | --- | --- |
| ROI surface area (mm²) | DL model | −0.4234 | [−25.4960, 24.6493] | 0.9704 | 1.0000 |
|  | Novice | 6.9483 | [−1.8733, 15.7699] | 0.1085 | 0.6508 |
|  | Expert 1 | −5.6976 | [−10.0179, −1.3773] | 0.0154 | 0.1076 |
| Peak flow amplitude (mL/s) | DL model | −0.0017 | [−0.5365, 0.5332] | 0.9946 | 1.0000 |
|  | Novice | 0.1298 | [−0.0502, 0.3097] | 0.1373 | 0.6865 |
|  | Expert 1 | −0.1156 | [−0.1959, −0.0354] | 0.0098 | 0.0786 |
| Stroke volume (mL) | DL model | −0.0129 | [−0.1304, 0.1046] | 0.8096 | 1.0000 |
|  | Novice | 0.0235 | [−0.0172, 0.0641] | 0.2244 | 0.8977 |
|  | Expert 1 | −0.0270 | [−0.0449, −0.0091] | 0.0078 | 0.0704 |

**Measurements.** Mean bias is the method-minus-reference difference averaged across 10 patients; negative values indicate lower measurements relative to the reference. For each patient and endpoint, the reference was the multi-expert reference (three experts, two sessions each). The novice and Expert 1 values were averaged across their two sessions within each patient; the DL model was evaluated once. Expert 1 contributed to the reference.

**Statistical analysis.** Each of the nine mean biases was tested against zero using a two-sided one-sample t-test on the patient-level differences (9 degrees of freedom). Unadjusted 95% confidence intervals were calculated using the t-distribution and the standard error of the mean difference. These intervals quantify uncertainty in the mean bias and are distinct from the 95% limits of agreement. Holm adjustment was applied across the nine mean-bias tests.

**Interpretation.** Expert 1 had negative mean biases with unadjusted p-values below 0.05 for all three biomarkers. No mean bias reached statistical significance after Holm correction at α = 0.05. Confidence intervals are unadjusted; exclusion of zero therefore corresponds to the unadjusted test. Nonsignificant results do not establish equivalence or close individual agreement. The t-based inference assumes approximately normally distributed patient-level differences and should be interpreted cautiously with 10 patients.

**Abbreviations.** CI, confidence interval; DL, deep learning; ROI, region of interest.
